# Hangover-Related Internet Searches Before and During the COVID-19 Pandemic in England: Observational Study

**DOI:** 10.2196/40518

**Published:** 2023-03-03

**Authors:** Eric Robinson, Andrew Jones

**Affiliations:** 1 Institute of Population Health Department of Psychology University of Liverpool Liverpool United Kingdom

**Keywords:** alcohol, COVID-19, hangover, Google Trends, social media, public health, online information, alcohol use, internet search

## Abstract

**Background:**

It is unclear whether heavy alcohol use and associated hangover symptoms changed as a result of the COVID-19 pandemic. Due to a lack of available accurate and nonretrospective self-reported data, it is difficult to directly assess hangover symptoms during the COVID-19 pandemic.

**Objective:**

This study aimed to examine whether alcohol-induced hangover-related internet searches (eg, “how to cure a hangover?”) increased, decreased, or remained the same in England before versus during the COVID-19 pandemic (2020-2021) and during periods of national lockdown. Secondary aims were to examine if hangover-related internet searches in England differed compared to a country that did not impose similar COVID-19 lockdown restrictions.

**Methods:**

Using historical data from Google Trends for England, we compared the relative search volume (RSV) of hangover-related searches in the years before (2016-2019) versus during the COVID-19 pandemic (2020-2021), as well as in periods of national lockdown versus the same periods in 2016-2019. We also compared the RSV of hangover-related searches during the same time frames in a European country that did not introduce national COVID-19 lockdowns at the beginning of the pandemic (Sweden). Hangover-related search terms were identified through consultation with a panel of alcohol researchers and a sample from the general public. Statistical analyses were preregistered prior to data collection.

**Results:**

There was no overall significant difference in the RSV of hangover-related terms in England during 2016-2019 versus 2020-2021 (*P*=.10; robust *d*=0.02, 95% CI 0.00-0.03). However, during national lockdowns, searches for hangover-related terms were lower, particularly during the first national lockdown in England (*P*<.001; *d*=.19, 95% CI 0.16-0.24; a 44% relative decrease). In a comparison country that did not introduce a national lockdown in the early stages of the pandemic (Sweden), there was no significant decrease in hangover-related searches during the same time period (*P*=.06). However, across both England and Sweden, during later periods of COVID-19 restrictions in 2020 and 2021, the RSV of hangover-related terms was lower than that in the same periods during 2016-2019. Exploratory analyses revealed that national monthly variation in alcohol sales both before and during the COVID-19 pandemic were positively correlated with the frequency of hangover-related searches, suggesting that changes in hangover-related searches may act as a proxy for changes in alcohol consumption.

**Conclusions:**

Hangover-related internet searches did not differ before versus during the COVID-19 pandemic in England but did reduce during periods of national lockdown. Further research is required to confirm how changes in hangover-related search volume relate to heavy episodic alcohol use.

**Trial Registration:**

Open Science Framework 2Y86E; https://osf.io/2Y86E

## Introduction

The COVID-19 pandemic brought about unprecedented changes to everyday life during 2020-2021. Restrictions imposed to reduce the spread of COVID-19, including national and local level lockdowns, are thought to have had substantial effects on population health and behavior. For example, there is some evidence that during the early stages of the pandemic, mental health symptoms increased sharply before decreasing as COVID-19 restrictions were removed [1,2].

Data on alcohol-related deaths in England and Wales from the Office for National Statistics appeared to show an increase during the period of April to September 2020 when compared to the years prior to the pandemic [3]. Based on available data in 2020, Public Health England [4] concluded that the prevalence of increasing-risk and higher-risk drinking increased when the pandemic began and then continued to be higher than previous years, which may explain an increase in alcohol-related deaths. During the early stages of the pandemic, several prospective survey studies of UK adults reported increases in heavy episodic drinking relative to prepandemic data. In one study of middle-age UK adults, the percentage of participants engaging in high-risk drinking (as defined by the Alcohol Use Disorder Identification Task) increased from 19% to 25% [5]. Likewise, in a different study of UK adults, heavy episodic drinking on a weekly basis increased from 10% to 17% before versus during the early stages of the pandemic [6].

However, a study of UK data found that during periods of COVID-19 restrictions, in which alcohol could not be purchased on trade (eg, in bars and restaurants), total household purchases of alcohol did not differ compared to 2015-2019 [7]. Likewise, an interrupted time-series analysis of repeat cross-sectional market research data from Scotland and England that accounted for both on-trade and off-trade purchasing [8] observed that overall, there was no change in estimated total units of alcohol consumed per week in the early stages of the pandemic. Furthermore, studies sampling German and UK adults during the early stages of the pandemic found that it was more common for participants to report that their alcohol-drinking behavior was unchanged or had reduced than it was to report that their alcohol consumption had increased [9,10]. Inconsistent findings may be due to methodological differences between studies. Although alcohol sales data are regarded as offering one of the most accurate measurements of population-level alcohol consumption [11], such data cannot be used to infer the amount consumed during individual drinking episodes. Conversely, self-reporting of alcohol consumption is prone to recall bias, and this is particularly problematic when large amounts of alcohol have been consumed [12].

One of the most commonly reported consequence of heavy episodic drinking is subsequently feeling “hungover” [13]. Experiencing hangovers is thought to increase the risk of a range of negative outcomes, ranging from increased mental health symptoms to neurological impairments [14]. Although studies have suggested potential changes to heavy alcohol consumption during the COVID-19 pandemic, to date, there has been limited research examining how the frequency of experiencing hangovers may have changed during the pandemic. In this research, we made use of internet search data to examine hangovers in the context of the COVID-19 pandemic.

Google Trends is a publicly accessible tool developed and provided by Google Inc [15]. The tool allows users to examine the frequencies of daily Google search terms based on geospatial criteria (eg, country level) and time periods of interest. Historical search results (per day) are normalized to represent a 0-100 scale. Each search “data point” is divided by the total searches of the region and time period it represents to compute relative popularity. The resulting numbers are scaled from 0-100 based on a topic’s proportion to all searches on all topics, otherwise known as relative search volume (RSV) [16]. Google Trends RSV data have been shown to be predictive of a range of objectively measured health-related behaviors. For example, increases in US Google Trends RSV data on HIV-related searches predict changes in HIV incidence rates at the state level [17]. RSV data for a range of symptom-level searches have been shown to predict regional outbreaks of infectious disease [16,18] and responses to public health campaigns (eg, increase in searches relating to smoking cessation during mass media smoking cessation campaigns) [19,20]. Studies using Google Trends data have been used to track changes in regional searches for COVID-19 symptoms and correlate with local infection rates [21], further suggesting that internet search data may be a useful surveillance tool to identify changes to health-related symptoms. In India, during the pandemic, alcohol was temporarily prohibited, and a study using Google Trends data found that searches relating to alcohol procurement and withdrawal increased after the introduction of the prohibition [22]. However, no research we are aware of has examined hangover-related searches in the context of the COVID-19 pandemic.

In this research, we made use of Google Trends data to explore for the first time whether the frequency of hangover-related search terms (eg, “how to cure a hangover”) changed before versus during the COVID-19 pandemic (2020-2021) and during periods of national lockdown restrictions in England.

## Methods

### Search Terms

#### Hangover-Related Searches

To identify terms commonly searched for when hungover, we consulted 10 UK–based alcohol researchers and 10 members of the general public. We checked commonly mentioned search terms that were used frequently enough to be recorded by Google Trends, resulting in the following terms: “hungover,” “hangover,” “how to cure a hangover,” “how to get rid of a hangover,” “what helps a hangover,” “hangover cure,” “cure for hangover,” “hangover remedy,” “hangover anxiety,” “hangover food,” and “alcohol poisoning” (quotation marks denote searches using the exact order of words). Searches for each term were conducted individually. Spelling mistakes for words related to hangover (eg, “hangovet”) were not considered as searches for these terms were too infrequent to be recorded by Google Trends.

#### Control Search

To rule out the possibility that any changes in hangover-related searches may simply be related to a general tendency for searches relating to help-seeking behavior to have increased or decreased, we conducted searches for a control search term. More specifically, we examined search terms for a medical symptom we presumed was unlikely to be a direct cause of COVID-19, unrelated to experiencing a hangover, and a relatively commonly experienced (but undesirable) state by most of the general population: experiencing trapped wind (aerophagia). Although a recent systematic review estimated that 2% of COVID-19 infection cases report abdominal pain [23], no research we were aware of has specifically identified trapped wind, aerophagia, belching (eructation), or farting (flatulence) as a commonly reported side effect of COVID-19 infection [23-25]. Search terms were selected to be similar in length to hangover-related searches (see Multimedia Appendix 1).

### Search Locations

Google Trends data were not consistently available in Scotland, Wales, and Northern Ireland for all search terms (ie, a large number of “0” RSV data, indicating very low search volume), and the dates of national lockdowns differed between UK countries. Therefore, in the primary analyses, searches were limited to the geographic region of England. If we found any evidence for changes in RSV for hangover-related search terms during any period of the pandemic (eg, change in RSV during lockdowns), we planned to repeat this analysis in a country that had not introduce similar measures. Sweden was selected as it is a predominantly English-speaking country that did not introduce a national lockdown or nonessential business closures during the early stages of the pandemic [26]. From November 2020 onward, Sweden did however introduce measures including the early closing of bars, limits on group sizes in bars and restaurants, and the closure of nonessential public services run by the state. We planned to use same English-language search terms as in our primary analyses; however, for many terms, there was no search volume in Sweden. As such, we limited the analyses to the terms “hangover” and “hungover” as well as their translation in Swedish (“baksmälla” and “bakfull,” respectively).

### Time Periods

To compare how frequently people made hangover-related searches during the COVID-19 pandemic in 2020-2021 versus before the pandemic, we collected RSV data from March 21, 2020 (when the World Health Organization declared COVID-19 a pandemic), to December 31, 2021, along with RSV data from March 21, 2016, to March 20, 2020 (prior to this period, an adjustment to Google Trends was made to improve the accuracy of search results). To examine the periods of COVID-19 national lockdown in 2020-2021 that were associated with a change in RSV, we compared the following periods: from March 23, 2020, to May 31, 2020 (first lockdown in England); from November 5, 2020, to December 6, 2020 (second lockdown); and from January 6, 2021, to March 28, 2021 (third lockdown), with the same dates during 2016-2019. To account for any potential differences in the number of weekdays versus weekends between years and the likelihood of hangover-related searches being more common on weekends, we recorded whether each day was a weekday versus weekend. Because RSV is a relative measure of search volume and therefore dependent on the specified time period searched, the time period specified in the searches represented the full time period of the study (as opposed to searching and extracting data separately for each individual prepandemic vs during pandemic or lockdown vs nonlockdown time periods).

### Planned Analyses

Google Trends data were extracted using the *gtrendR* R package, in combination with modified code by Dyachenko [27], allowing for daily estimates of RSV over long periods of time. Google Trends provides daily estimates if the search period is <9 months; otherwise, it provides weekly (if the search period is between 9 months to 5 years) or monthly (if the search period is >5 years) estimates. Dyachenko [27] modified *gtrendR* to query daily estimates on a monthly time frame, before weighting these estimates against monthly data for the whole time frame. Some RSV data had considerably more variability than others, and some had a considerable number of 0s—indicative of very low (inestimable) search volume on days. As such, to compare prepandemic versus during pandemic search volume, we conducted Yuen trimmed means *t* tests to negate the impact of increased 0s, as well as standard nonparametric tests to clarify findings. To determine the size of the effect, we used a robust estimation of Cohen *d*, based on the trimmed means [28]. For complex comparisons with multiple factors (eg, lockdown time periods before vs during the pandemic, weekdays vs weekends, and their interaction), we used trimmed ANOVAs (*t2way* and *t3way* from the *WRS2* R package). Trimmed ANOVAs performed via *WRS2* do not compute degrees of freedom since an adjusted critical value is used. However, we report degrees of freedom from a standard ANOVA. To examine whether hangover-related RSV was related to “banana bread” RSV, we correlated the 2 types of search data (daily RSVs). Data processing and analyses were conducted in R software (R Foundation for Statistical Computing), using the *WRS2* and ggstatsplot packages. To account for multiple comparisons being conducted, the statistical significance was set at *P*<.01 for all analyses and all tests were two-tailed.

### Preregistration

The preregistered analysis strategy, analysis code, and data set are available on the web (Open Science Framework 2Y86E). For deviations from the preregistered protocol, see Multimedia Appendix 1. Given the large sample sizes (the combination of search terms for the large number of days before and during the pandemic), primary analyses were more than adequately powered (eg, for the main effects of comparing hangover-related activity before vs during the pandemic, we were powered to detect very small effects [*d*s<0.1] with 90% statistical power).

### Public or Patient Involvement

There was public involvement in the identification of search terms used in the study.

### Ethical Considerations

The research was exempt from institutional ethics review as there was no new collection of data from human or nonhuman research subjects.

## Results

### Comparison of Hangover-Related Search Activity From Before the Pandemic to During the Pandemic

**Figure 1:**
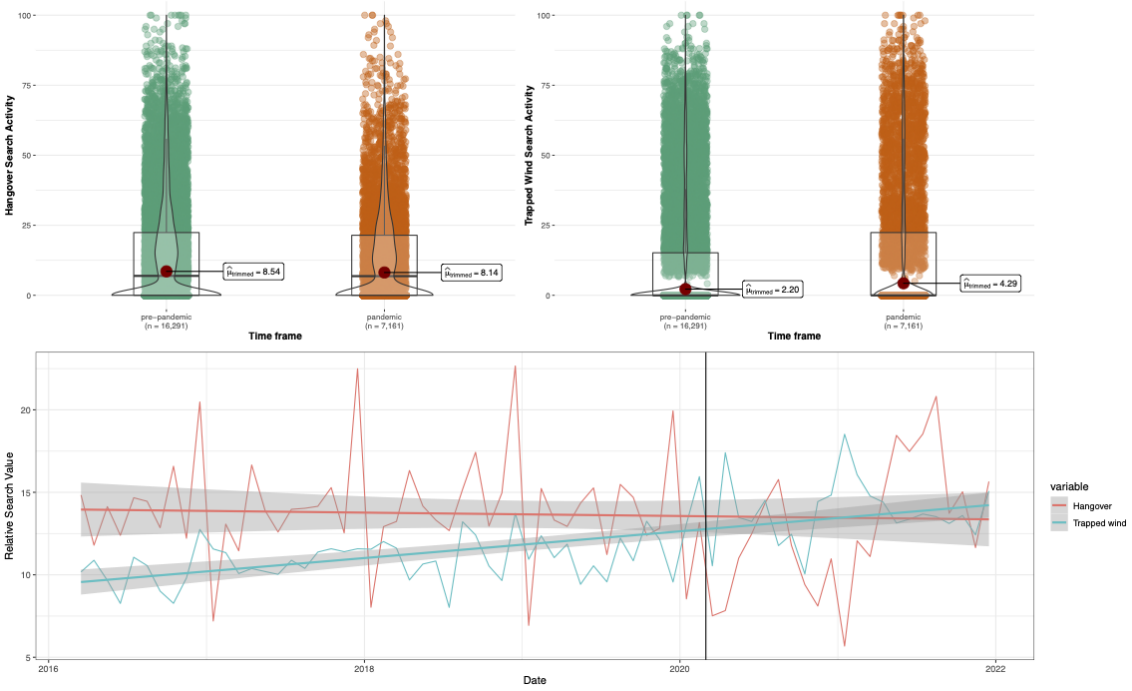
Prepandemic versus during pandemic search activity for hangovers and trapped wind. Top left panel: hangover-related search activity; top right panel: trapped wind–related search activity; bottom panel: hangover and trapped wind–related search activity over time (thick black line represents the start of the pandemic).

Overall, there was no significant difference in hangover-related RSV during the pandemic versus before the pandemic (Yuen *t*_8670_=1.63; *P*=.10; robust *d*=0.02, 95% CI 0.00-0.03). However, there was a small and significant increase in searches related to the control search term, trapped wind (Yuen *t*_6416_=7.21; *P*<.001; robust *d*=.08, 95% CI 0.06-0.10; see Figure 1).

### Changes in Hangover-Related Searches: Lockdown Periods

#### Lockdown Period 1 (March 23 to May 31, 2020)

The main effects of prepandemic versus pandemic periods and weekday versus weekend were significant and subsumed under a significant interaction (*F*_1,3076_=28.05; *P*<.001). RSV was greater in nonpandemic periods (trimmed mean=9.32) versus pandemic periods (trimmed mean=5.20; robust *d*=.19, 95% CI 0.16-0.24; equating to a 44% relative decrease) but also on weekends (trimmed mean=19.83) versus weekdays (trimmed mean=5.25; robust *d*=0.51, 95% CI 0.48-0.55). The interaction was the result of the difference in RSV between pandemic and prepandemic time periods being smaller on weekdays (difference=2.41, 95% CI 1.26-3.56) versus weekends (difference=12.59, 95% CI 8.99-16.17).

#### Lockdown Period 2 (November 5 to December 6, 2020)

The main effects were significant and subsumed under a significant weekday/weekend vs pandemic/nonpandemic interaction (*F*_1,1184_=22.32; *P*<.001). RSV was greater in nonpandemic periods (trimmed mean=7.77) versus pandemic periods (trimmed mean=4.79; robust *d*=.18, 95% CI 0.11-0.23; equating to a 38% relative decrease) but also on weekends (trimmed mean=19.00) versus weekdays (trimmed mean=4.41; robust *d*=0.57, 95% CI 0.53-0.63). The interaction was the result of the difference in RSV between pandemic and prepandemic time periods being smaller on weekdays (difference=1.26, 95% CI –0.29 to 2.81) versus weekends (difference=14.70, 95% CI 9.30-20.10).

#### Lockdown Period 3 (January 6 to March 28, 2021)

The main effects were significant and subsumed under a significant weekday/weekend vs pandemic/nonpandemic interaction (*F*_1,36040_=6.97; *P*=.009). RSV was greater in nonpandemic periods (trimmed mean=6.20) versus pandemic periods (trimmed mean=5.29; robust *d*=.05, 95% CI 0.00-0.09; equating to a 15% decrease) but also on weekends (trimmed mean=16.68) versus weekdays (trimmed mean=3.41; robust *d*=0.58, 95% CI 0.55-0.60). The interaction was the result of a difference in RSV between pandemic and prepandemic time periods being smaller on weekdays (difference=0.38, 95% CI –0.50 to 1.26) versus weekends (difference=4.88, 95% CI 1.64-8.12).

### Comparison Country: Sweden During England Lockdown Dates

#### Dates of Lockdown Period 1 in England (March 23 to May 31, 2020)

During this period, there was no significant COVID-19 restrictions in Sweden. The main effect of prepandemic versus pandemic periods was not significant (trimmed mean before the pandemic=7.09; trimmed mean during the pandemic=5.34; *F*_1,1068_=3.67; *P*=.06). There was a significant difference between weekend (trimmed mean=15.95) versus weekday searches (trimmed mean=4.39; *F*_1,1068_=55.86; *P*=.001). There was no significant interaction (*F*_1,1068_=2.31; *P*=.12).

#### Dates of Lockdown Period 2 in England (November 5 to December 6, 2020)

During this period, some COVID-19 restrictions were introduced in Sweden. The main effects of time (*F*_1,428_=21.13; *P*<.001) and weekday/weekend (*F*_1,428_=26.68; *P*<.001) were subsumed under a significant interaction (*F*_1,428_=10.55; *P*<.001). The RSV was significantly greater before the pandemic (trimmed mean=8.25) compared to during the pandemic (trimmed mean=3.77; robust *d*=.28, 95% CI .28-.45). It was also greater on weekends (trimmed mean=15.95) versus weekdays (trimmed mean=4.63; robust *d*=.46, 95% CI .37-.56). The interaction was the result of a difference in RSV between pandemic and prepandemic time periods being smaller on weekdays (difference=2.57, 95% CI –0.09 to 5.24) versus weekends (difference=14.97, 95% CI 7.84-22.08).

#### Dates of Lockdown Period 3 in England (January 6 to March 28, 2021)

During this period, there were a number of COVID-19 restrictions in Sweden. There was a main effect of time (*F*_1,1308_=11.72; *P*<.001) and weekday/weekend (*F*_1,1308_=105.98; *P*<.001). The RSV was greater before the pandemic (trimmed mean=6.82) compared to during the pandemic (trimmed mean=5.13; robust *d*=.12, 95% CI .02-.21). It was also greater on weekends (trimmed mean=14.77) versus weekdays (trimmed mean=3.98; robust *d*=.57, 95% CI .52-.62). There was no significant interaction (*F*_1,1308_=6.44; *P*=.012).

#### Sweden Before Versus During the Pandemic

As in the main analyses for England, we also examined whether hangover-related searches differed across the total prepandemic versus pandemic time periods. Similar to England, hangover-related RSV was somewhat reduced during the pandemic versus before the pandemic (Yuen *t*_3516_= 2.20; *P*=.03; robust *d*=0.04, 95% CI 0.01-0.06), but this difference was very small and not statistically significant based on our preregistered analysis plan.

### Unplanned Analyses

#### Trapped Wind and COVID-19 Cases

As we unexpectedly observed an increase in trapped wind searches during versus before the pandemic, we explored whether this change in search volume may have been caused by the spread of COVID-19. We extracted daily estimates of verified COVID-19 cases from January 30, 2020, to December 31, 2022, from the UK Government COVID-19 database and examined the association between daily trapped wind searches and the number of daily cases reported. There was a small positive association (*r*_650_=.079, 95% CI .002-.155; *P*=.04), but this was not statistically significant based on our preregistered α of *P*<.01.

#### Relationship of National Hangover-Related Searches and Alcohol Sales

To explore the possibility that the frequency of hangover-related searches acts as a proxy for alcohol consumption, we were able to obtain monthly alcohol sales data in the form of alcohol duty within the United Kingdom. We correlated this monthly data against the average RSV across our hangover-related search terms for the same months from March 2016 to December 2021 and found a significant positive correlation both before and during the pandemic (*r*_68_=.47; *P*<.01). See Multimedia Appendix 1 for full analyses and graphical representation.

## Discussion

### Principal Findings

The objective of this research was to use Google Trends data to explore whether the frequency of hangover-related search terms (eg, “how to cure a hangover”) changed before versus during the COVID-19 pandemic (2020-2021) and during periods of national lockdown restrictions in England. The frequency of hangover-related internet search terms was similar in the years before (2016-2019) versus during (2020-2021) the COVID-19 pandemic in England. However, during periods of national lockdown, there was a significant decrease in the frequency of hangover-related internet search terms when compared to the same periods in the years before the pandemic. This decrease in hangover-related searches was observed on both weekdays and weekends.

Studies estimating changes to population-level alcohol consumption patterns have been limited to inferring consumption from household alcohol sales data or have relied on retrospective participant self-reports of alcohol consumption [5,7,8,10]. These studies have tended to produce mixed results on alcohol use during the pandemic. An interpretation of this study’s findings is that for a significant proportion of the population, heavy episodic alcohol use reduced during periods of national lockdowns, and this caused a decrease in hangover-related internet searches. A strength of this research is that our measurement of hangover-related internet search activity is not reliant on retrospective participant self-reports. Consistent with the observation that alcohol consumption tends to be heavier and more frequent on weekends versus weekdays, in this research, we found that hangover-related searches were more common on weekends than weekdays both before and during the COVID-19 pandemic [29,30]. However, prior to this work, no research we are aware of has examined whether changes in hangover-related search activity predict changes in objectively measured alcohol consumption. To explore whether hangover-related searches may act as a proxy measure of alcohol consumption, we conducted exploratory analyses and found that monthly alcohol sales and the frequency of hangover-related searches were significantly correlated both before and during the COVID-19 pandemic.

In addition to being a likely proxy measure of heavy episodic alcohol drinking, changes to hangover-related search activity could also occur due to changes in help-seeking motivation (ie, being less likely to search for hangover cures due to a lack of motivation). There is some evidence that lockdown measures in the United Kingdom were associated with worsening mental health, stress, and loneliness [1,31], which may have theoretically affected how motivated people were to search for health-related information. In addition, changes to hangover search activity could be in part caused by other changes to lifestyle patterns that resulted in less need to treat hangover symptoms (eg, increased number of people not working due to furlough or working from home), although we observed decreases in hangover-related searches for both weekday and weekend lockdown days.

The severity of hangover symptoms is likely to differ based on a range of factors and population characteristics, including genetic differences in susceptibility to the pharmacological effects of alcohol, level of alcohol dependence, and amount of alcohol consumed during drinking episodes [32-34]. In addition, the likelihood of searching for hangover-related terms may differ based on population demographics (eg, searching being more common among people experiencing a hangover for the first time). Therefore, a limitation of the study is that the available data do not allow us to characterize whether observed changes in hangover-related searches differ across population demographics. A further limitation of this research is that the observational approach used does not allow us to make causal inferences. Associations observed between lockdown periods in England and hangover-related searches may be affected by unmeasured variables that also changed as a result of the pandemic. In an attempt to address this concern, we examined the search volume for an unpleasant bodily complaint that we reasoned the search activity should not have changed as a result of the COVID-19 pandemic: trapped wind. Unlike the decreases we observed for hangover-related searches, we unexpectedly found that searches for trapped wind were more frequent during the pandemic compared to before the pandemic. It may be the case that other gastrointestinal symptoms associated with COVID-19 (eg, abdominal pain and nausea) are routinely misinterpreted as trapped wind and thus explains the increase in search volume. There was a weak positive (nonsignificant) association whereby trapped wind searches were more frequent during days in which a higher number of COVID-19 cases were reported.

A strength of this research is our use of a comparison country with fewer COVID-19 restrictions during the early stages of the pandemic. Sweden did not implement any national lockdown measures at the beginning of the pandemic, and during this period, unlike the RSV data for England, we found no evidence of a significant decrease in the frequency of hangover-related search terms. This increased confidence in our conclusions that reductions to hangover-related searches likely occurred as a result of COVID-19 restrictions. From November 2020 onward, Sweden introduced restrictions that limited social movement (eg, including the early closing of bars and limits on group sizes in bars and restaurants). In line with this, during the November to December 2020 and January to March 2021 periods, we observed a decrease in hangover-related search frequency for both England and Sweden. A further strength of this research is that we were able to compare data in the pandemic years (2020-2021) with similar data spanning 2016-2019. Although we selected search terms on the basis of consultation with alcohol researchers and the general public to capture likely terms searched for when hungover and we present data suggesting that hangover-related searches and alcohol sales are correlated, as discussed, we do not know to what extent changes in internet search–related activity definitively predict changes in heavy drinking patterns. It would therefore be valuable for future research to further examine the associations between hangover (or other alcohol-related) internet searches, alcohol sales, and heavy alcohol consumption patterns. As has been suggested for other topics of public health importance [16], it may be the case that internet search–based activity can be used to detect population-level changes in alcohol-related behavior, and this therefore could be a valuable methodology for future research on hangovers and alcohol use. Some data indicate that alcohol-related deaths may have increased during the pandemic [3] and a proportion of the population may have increased their frequency of heavy episodic “binge” drinking in the early stages of the pandemic [5]. This highlights that alcohol consumption and misuse remain an important public health issue, and future research is needed to continue to monitor and understand changing patterns in alcohol consumption and their effects on public health.

### Conclusions

Hangover-related internet searches did not differ before versus during the COVID-19 pandemic in England but did reduce during periods of national lockdown.

## Appendix

Multimedia Appendix 1 Supplementary material.

## Abbreviations

RSV: relative search volume

## Data Availability

The preregistered analysis strategy, analysis code, and data set are available on the web (Open Science Framework 2Y86E; https://osf.io/2Y86E).
