# Supplementary material for: Hangover-Related Internet Searches Before and During the COVID-19 Pandemic in England: Observational Study
Source: JMIR Form Res. 2023 Mar 3;7:e40518. doi: 10.2196/40518 (PMC9994424; doi:10.2196/40518)
Supplement: Multimedia Appendix 1 [file formative_v7i1e40518_app1.docx]

Online Supplementary Materials: Robinson & Jones, 2022

Trapped wind related search terms

“trapped wind”

“wind trapped”

“how to cure trapped wind”

“how to get rid of trapped wind”

“what helps trapped wind”

“trapped wind cure”

“cure for trapped wind”

“trapped wind remedy”

“trapped wind cause”

“help trapped wind”

“medicine for trapped wind”

Changes from pre-registered analysis plan

1) In our original pre-registered analyses we included a series of planned searches relating to baking banana bread. This was included as a humorous / light take on what people were likely to be making more internet searches for during the pandemic, as we intended to submit this work to a journal with a special edition that requires authors to approach submitted research with a humorous / light hearted angle. We do not include data on these searches and analyses (data available on request from the authors) as we did not believe it to be appropriate for this journal. The findings showed that RSV increased for banana bread baking / recipe searches during periods of national lockdown in England this increase did not correlate with hangover-related searches. See the original pre-registration in full on **the Open Science Framework (**DOI 10.17605/OSF.IO/2Y86E**) at** <https://osf.io/2y86e/>).

2) In the pre-registered analysis plan we intended to examine whether specific days during the pandemic were associated with changes in hangover-related search activity (e.g. reopening of pubs in 2020, final removal of all COVID restrictions in 2021). However, google trends data for historic dates was not available in sufficient detail (e.g. hourly data) in order to conduct statistical analyses (i.e. RSV data was only available for individual days and this would result in a single data point). This same issue prevented us exploring using join point analyses to examine time trends for RSV (Join point regression identifies changes in trend data (e.g. is the relationship between time and search volume stable over time, or are their multiple, different trend-slopes).

3) To explore the possibility that alcohol sales may relate to frequency of hangover-related searches we were able to obtain monthly alcohol sales data in the form of alcohol duty within the UK. The UK Government publishes this data monthly (https://www.gov.uk/government/statistics/alcohol-bulletin). We correlated this monthly data against the average RSV across our hangover-related search terms for the same month. We did this from March 2016 – December 2021, ensuring we had similar coverage of time periods from our RSV analysis. Importantly, we note that there was a correlation between average monthly hangover-related search activity and alcohol duty from sales (r(68) = .47, P < .01: see Figure below). Furthermore, the strength of this correlation was robust regardless of whether the data was taken from pre-pandemic (r(46) = .56, P < .001) or during the pandemic (r(20) = .46, P = .029).

**
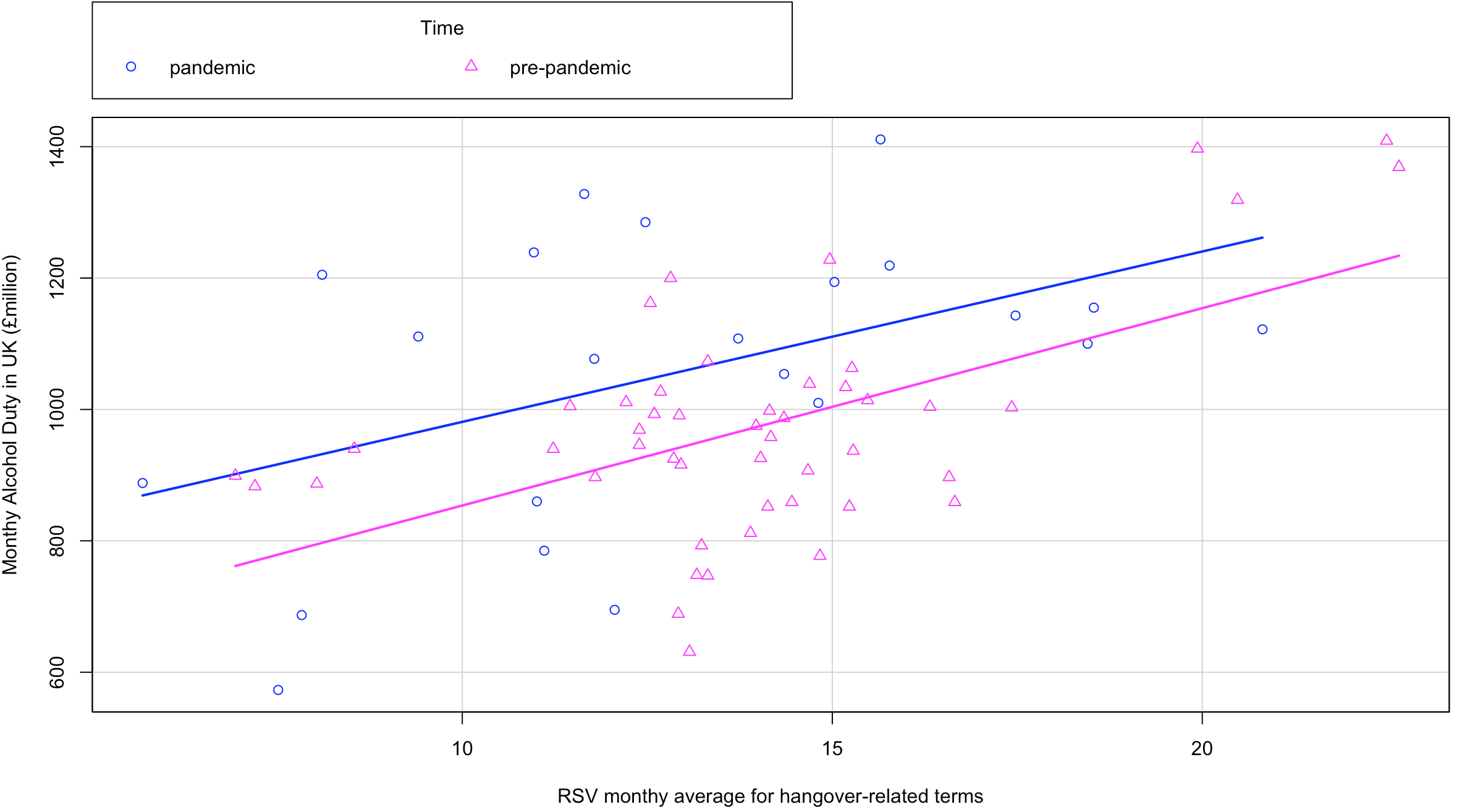
**

Notably, when correlating average searches for trapped-wind related terms the correlation with monthly alcohol duty was negligible and non-significant (r(68) = .05, *P* = .635: see Figure below).

**
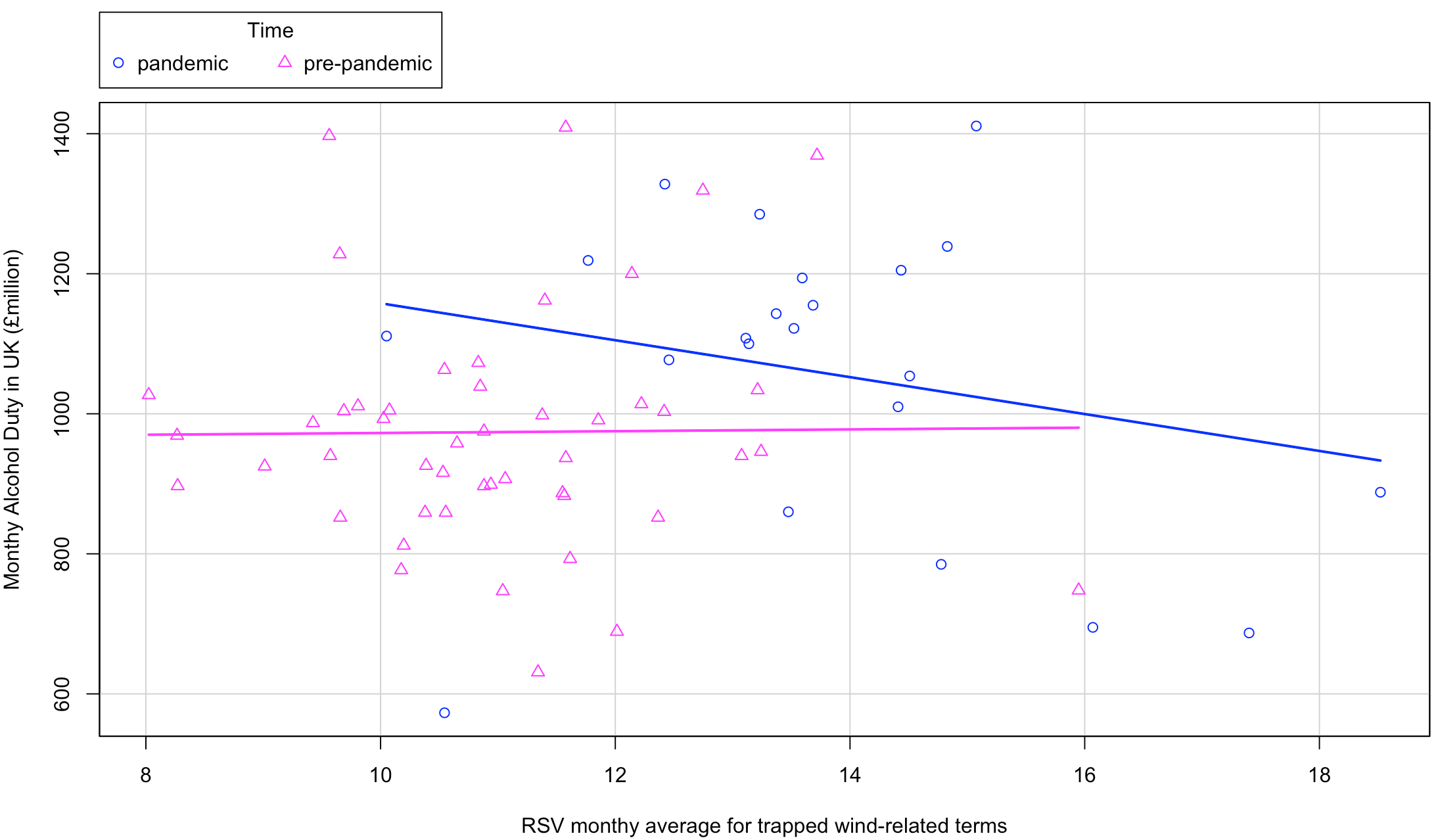
**
